# Work Fatigue Profiles: Nature, Implications, and Associations With Psychological Empowerment

**DOI:** 10.3389/fpsyg.2020.596206

**Published:** 2020-11-26

**Authors:** Ann-Renée Blais, Nicolas Gillet, Simon A. Houle, Caitlin A. Comeau, Alexandre J. S. Morin

**Affiliations:** ^1^Department of National Defence, Ottawa, ON, Canada; ^2^Département de Psychologie, Université de Tours, Tours, France; ^3^Institut Universitaire de France (IUF), Paris, France; ^4^Substantive-Methodological Synergy Research Laboratory, Concordia University, Montreal, QC, Canada

**Keywords:** work fatigue, latent profiles, psychological empowerment, satisfaction, turnover

## Abstract

The present study examined the distinct configurations, or profiles, taken by work fatigue dimensions among samples of military (*n* = 1,436) and civilian (*n* = 2,477) employees. We also tested profile similarity across these two samples of employees. In addition, this research documented the relations between the identified work fatigue profiles, one predictor variable (psychological empowerment), and a series of attitudinal outcomes (job satisfaction, career satisfaction, and turnover intentions) among military employees. Six profiles of employees characterized by different levels of global and specific (emotional, physical, and mental) work fatigue were identified using latent profile analyses: *Low Fatigue, Physically and Emotionally Depleted, Emotionally Depleted, Globally and Mentally Depleted, Globally and Emotionally Depleted*, and *Balanced*. In both samples, employees corresponding to the *Balanced* profile displayed average levels of global and specific work fatigue. However, this profile slightly differed across sample, as indicated by the observation of work fatigue levels that were slightly higher among the military than among civilians. Militaries’ perceptions of psychological empowerment were significantly related to their likelihood of belonging to all profiles. In turn, militaries’ career satisfaction, job satisfaction, and turnover intentions were also found to differ as a function of profile membership.

## Introduction

Work fatigue is typically defined as a reduction of one’s functional capacity due to extreme tiredness (Frone and Tidwell, 2015) and has long been recognized as a precursor of a wide variety of undesirable outcomes for the organization (e.g., higher levels of turnover intentions; Cai et al., 2018) and the employee (e.g., lower sleep quality and quantity; Frone and Blais, 2019). Frone and Tidwell (2015) showed that employees presenting high levels of work fatigue displayed lower job satisfaction, psychological health, physical health, and organizational commitment, coupled with accrued turnover intentions and difficulties to relax after work. Work fatigue is damaging both psychologically and physically, leading to less efficient work recovery, negative work attitudes, and health-related difficulties (Hobfoll, 1989; Shirom and Melamed, 2006).

Despite abundant research (Frone, 2016; Barling and Frone, 2017) supporting the negative consequences associated with the various components of work fatigue (physical, mental, and emotional; Frone and Tidwell, 2015), their combined impact remains understudied. To better understand this combined impact, two complementary approaches can be used. Variable-centered analyses, designed to assess how variables (such as fatigue components) relate to other variables, are able to test for interactions among these variables (i.e., to verify if the effects of a predictor differ as a function of a moderator variable). Unfortunately, variable-centered analyses assume that the observed associations generalize equally to all members of the sample, and tests of variable-centered interactions are almost impossible to interpret meaningfully when they involve multiple predictors (i.e., more than 2 or 3) and/or non-linearity.

Alternatively, person-centered analyses are explicitly designed to identify subpopulations presenting differentiated configurations on multiple indicators (such as work fatigue dimensions) in order to assess how these configurations relate to various predictors and outcomes. No person-centered studies have yet, to the best of our knowledge, examined the various configurations taken by work fatigue components within specific employees (Frone and Tidwell, 2015). The present study addresses this limitation by documenting the work fatigue configurations that best characterized distinct profiles of employees. More importantly, these profiles are estimated while considering the multidimensionality of work fatigue through the joint consideration of workers’ levels of global and specific (emotional, physical, and mental) work fatigue (e.g., Isoard-Gautheur et al., 2018).

To ascertain the meaningfulness of the profiles, this study also systematically investigates whether these profiles generalize across military and civilian samples (Meyer and Morin, 2016; Morin et al., 2016d). Finally, the construct validity of the profiles will also be examined by considering their relations with psychological empowerment (PE), as a determinant, and a variety of outcomes (turnover intentions, job satisfaction, and career satisfaction) (Muthén, 2003; Marsh et al., 2009). Given that person-centered results tend to be more naturally aligned with managers tendency to think about their employees as corresponding to different categories (Zyphur, 2009; Morin et al., 2011b), our findings are likely to have important implications for practice. For instance, results from the predictive analyses should help us to identify the profiles with the most desirable, or undesirable, configuration from an outcomes perspective, and to document the possible role of PE as an actionable lever to decrease the likelihood of less desirable work fatigue profiles. Furthermore, evidence of generalizability across samples would reinforce the idea that the profiles tap into some core psychological phenomenon for which generic interventions could be devised in order to differentially manage, support, select, or even promote employees based on their profiles irrespective of employment type.

### Multidimensionality: Global Versus Specific Levels of Work Fatigue

A comprehensive representation of work fatigue should encompass the emotional, physical, and mental facets of this construct (Frone and Tidwell, 2015). More precisely, Frone and Tidwell (2015, p. 274) note that “physical work fatigue represents extreme physical tiredness and reduced capacity to engage in physical activity that is experienced during and at the end of the workday. Mental work fatigue represents extreme mental tiredness and reduced capacity to engage in cognitive activity that is experienced during and at the end of the workday. Emotional work fatigue represents extreme emotional tiredness and reduced capacity to engage in emotional activity that is experienced during and at the end of the workday.” However, despite this multidimensionality, some research results have suggested that employees could experience work fatigue holistically as a single global dimension (Frone, 2016). Such a global representation of work fatigue is supported by the generally high correlations reported among ratings of physical, mental, and emotional work fatigue (Frone et al., 2018), and the demonstration of stronger associations with covariates (i.e., predictors and outcomes) when work fatigue is defined as a higher-order dimension (Shirom and Melamed, 2006). In contrast, research has also supported the conceptually-distinct nature of the emotional, physical, and mental facets of work fatigue via the demonstration of differentiated covariate associations (Frone and Blais, 2019). For instance, Frone and Tidwell (2015) showed that physical job demands were significantly and positively related to physical work fatigue and weakly related to mental and emotional work fatigue. Mental job demands were also significantly and positively related to mental work fatigue and weakly related to physical and emotional work fatigue. Moreover, role conflict was associated with higher levels of emotional and mental work fatigue, but not significantly related to physical work fatigue. In terms of outcomes, physical work fatigue was negatively related to physical health, whereas mental and emotional physical work fatigue was not significantly related to this outcome. In contrast, the negative effects of emotional work fatigue on mental health and job satisfaction were stronger than those of physical and mental work fatigue.

A third possibility also exists. Indeed, it is also possible that global levels of work fatigue could co-exist with specific levels of emotional, physical, and mental work fatigue left unexplained by this global level. Higher-order results reported by Shirom and Melamed’s (2006) lend tentative support to this possibility by demonstrating that emotional, physical, and mental work fatigue are conceptually-related facets of an overarching factor, and yet retain specificity unexplained by this global factor. However, additional studies are needed to confirm that enough specificity exists in the emotional, physical, and mental dimensions once global levels of work fatigue are considered (Morin et al., 2016b, 2017). This remaining question can be addressed, psychometrically, using bifactor models. Indeed, bifactor models directly estimate the global (G-) factor (global work fatigue) from participants’ ratings of all indicators, as well as a series of orthogonal specific (S-) factors, also estimated directly from participants’ ratings of the indicators assumed to reflect the *a priori* dimensions (emotional, physical, and mental) (Chen et al., 2006). Estimated in this manner, the S-factors directly reflect the extent to which specific levels of emotional, physical, and mental fatigue deviate from global levels of work fatigue. Although never applied to work fatigue, this approach has often been found to match the structure of conceptually similar burnout measures (Hawrot and Koniewski, 2018; Isoard-Gautheur et al., 2018).

### Work Fatigue Profiles

Person-centered analyses are specifically designed to identify qualitatively distinct subpopulations of workers characterized by distinct configurations of work fatigue components (Meyer and Morin, 2016). Importantly, when applying person-centered analyses to indicators known to present a global/specific bifactor structure, it is essential to rely on profile indicators allowing for a proper disaggregation of these global (global levels of work fatigue) and specific (unique levels of emotional, physical, and mental work fatigue) components (Morin et al., 2016b, 2017). Indeed, failure to properly unpack these two layers of complexity has been shown to result in the erroneous estimation of profiles characterized by matching levels across indicators, thereby reflecting only global levels of work fatigue and ignoring meaningful specificities located at the subscale level (Morin et al., 2016b, 2017). In the present research, we examine how global and specific (emotional, physical, and mental) levels of work fatigue combine together among distinct subpopulations of workers. Lacking guidance from previous person-centered studies of work fatigue profiles (Frone and Tidwell, 2015), precise hypotheses cannot be formulated regarding the expected number and structure of work fatigue profiles. However, as described in the following sections, results from studies on conceptually-related constructs can still guide the present study.

#### Person-Centered Studies of Employee Burnout

First, past person-centered studies (Leiter and Maslach, 2016; Berjot et al., 2017; Guidetti et al., 2018; Laverdière et al., 2018; Portoghese et al., 2018; Schult et al., 2018) have examined the combined effects of burnout components (e.g., Maslach et al., 1996; Demerouti et al., 2003). Unfortunately, some of these previous studies have relied on a combination of burnout components and other variables as profile indicators (work engagement: Mäkikangas et al., 2017; workaholism and work engagement: Innanen et al., 2014; work engagement, job satisfaction, and workaholism: Mäkikangas et al., 2015; psychological distress and satisfaction with life: Laverdière et al., 2018), making it impossible to isolate the effects of burnout components in the definition of the profiles. In addition, many of these investigations have solely focused on global levels of burnout, making it impossible to consider to unique role played by burnout components.

Among the few exceptions, Berjot et al. (2017) identified psychologists’ burnout profiles based on emotional exhaustion, depersonalization, and personal accomplishment. They identified four profiles: (1) High risk of burnout (22.9%) across dimensions; (2) Risk of burnout through low personal accomplishment (27.1%); (3) Risk of burnout through emotional exhaustion (28.0%); and (4) No risk of burnout (22.0%) across dimensions. However, this study is limited by relying on a sample of psychologists, so that additional studies are needed to generalize these findings to other occupations.

Another study by Leiter and Maslach (2016), identified burnout profiles within health-care workers. In two distinct samples, their results revealed five distinct profiles: (1) Burnout: Moderate to high emotional exhaustion, cynicism, and professional inefficacy; (2) Disengaged: High cynicism, moderate to high emotional exhaustion, and moderate professional inefficacy; (3) Overextended: High emotional exhaustion, and moderate cynicism and professional inefficacy; (4) Ineffective: High professional inefficacy, and low to moderate cynicism and emotional exhaustion; and (5) Engagement: Low levels of emotional exhaustion, cynicism, and professional inefficacy. However, like the profiles identified by Leiter and Maslach (2016); Berjot et al. (2017) relied on typical scale scores, thus failing to consider global and specific levels of burnout (presenting similarity to the emotional, mental, and physical components of work fatigue).

#### Person-Centered Research on Other Related Dimensions

Other person-centered studies focusing on well-being, psychological health, and recovery experiences are also informative. In a study of psychological well-being at work, Morin et al. (2017) provided evidence for the adequacy of a bifactor solution, and relied on factor scores from this solution to identify employees’ profiles of well-being. Their results revealed four profiles: (1) Normative: corresponding to a majority of employees presenting average levels of well-being across dimensions (involvement, interpersonal fit, recognition, thriving, and competence); (2) well-integrated (4.8%); (3) intrinsically-driven (29.2%); and (4) ill-adjusted extrinsically-driven (6.6%). Relying on a similar approach to study psychological health (encompassing distress and well-being) at work, Morin et al. (2017) identified five profiles: (a) Normative (61.0%); (b) harmoniously-distanced (12.3%); (c) Adapted (11.1%); (d) stressfully-involved (14.3%; similar to the ill-adjusted extrinsically-driven profile); and (e) Flourishing (1.38%; similar to the well-integrated profile).

Numerous studies also considered work recovery experiences via the adoption of a person-centered perspective (e.g., Siltaloppi et al., 2012; Huhtala et al., 2017; Perko et al., 2017). The nature, number, and range of psychological constructs assumed to be part of employees’ work recovery experiences considered across these studies is quite large (psychological detachment, rumination, overcommitment, need for recovery, problem-solving pondering, work interruption in non-work behaviors, relaxation, mastery, control, etc.). Furthermore, these studies have also relied on a variety of samples (mixed employees samples, managers, school psychologists, etc.), methods (cross-sectional and longitudinal), and covariates. This variety makes it particularly hard to achieve a clear integration of these previous findings. Yet, despite these important differences, the results converge on the following profiles: (a) *High Recovery*; (b) *Moderately High Recovery*; (c) *Moderately Low Recovery*; and (d) *Low Recovery.*

#### A Person-Centered Perspective of Work Fatigue

What stands out from the aforementioned results is the high level of similarity across studies and constructs, and the fact that most authors failed to consider the coexistence of global and specific components of work burnout. This limitation might have led them to identify similarly-shaped profiles differing mainly in terms of global burnout (i.e., referred to as *level*-differentiated profiles relative to *shape*-differentiated profiles; Morin and Marsh, 2015) and to ignore potentially critical distinctions related to specific burnout components. Indeed, when relying on first-order, rather than bifactor, factor scores Morin et al. (2016b, 2017) identified profiles presenting almost pure quantitative (*level*) differences. In the present research, we adopt the approach advocated by these authors to extend to the work fatigue area their results obtained in research on employee psychological health and well-being.

Lacking prior guidance from work fatigue research more specifically (e.g., Frone and Tidwell, 2015) and from studies of similar constructs in which the multidimensional global/specific nature of employees’ ratings were properly disaggregated, we leave as an open research question the structure and number of profiles that will best reflect employees’ work fatigue configurations. Nevertheless, in alignment with the consistency of the findings obtained in person-centered research focusing on well-being, psychological health, and burnout (Leiter and Maslach, 2016; Morin et al., 2016b, 2017), it seems reasonable to assume that one of these profile will display a Balanced configuration (presenting average levels of work fatigue across indicators). Likewise, other profiles should display a Depleted (globally high levels of work fatigue across indicators) and a Low Fatigue (presenting globally low levels of work fatigue across indicators) configuration. Conversely, in accordance with a subset of *shape*-differentiated profiles obtained in burnout research (Leiter and Maslach, 2016; Berjot et al., 2017) and with Morin et al. (2016b, 2017) findings, it is also reasonable to expect the identification of additional profiles characterized by more differentiated configurations across dimensions. For example, a Globally and Emotionally Depleted profile (high global levels of work fatigue and emotional work fatigue, and average levels of physical and mental work fatigue), similar to Leiter and Maslach’s (2016) Overextended profile, might be identified.

### Profile Similarity Across Samples

Many have noted that a core aspect of the construct validation process of person-centered solutions involves the verification of the extent to which a profile solution can be reliably replicated across samples (e.g., Solinger et al., 2013; Meyer and Morin, 2016; Morin et al., 2016d). In this study, we address this issue by examining whether the identified work fatigue profiles generalize across military and civilian employees.

Previous research has shown variations in work fatigue as a function of job settings (Rupert and Kent, 2007), making it important to verify whether profiles generalize to the military and civilian contexts. For instance, McCormack et al. (2018) showed that workers from the private sector experienced less exhaustion than non-private employees. More generally, research shows that workplace characteristics, such as job design (e.g., autonomy and variety) or emotional demands, are significantly associated with employees’ work fatigue (Frone et al., 2018; Frone and Blais, 2019). For instance, Frone and Tidwell (2015) showed that exposure to different types of job demands (mental, emotional, and physical) displayed a well-differentiated pattern of association to distinct components of work fatigue (mental, emotional, and physical).

Military and civilian jobs clearly differ in character and requirements (Oprins et al., 2018). More specifically, military workers continuously face a range of stressful conditions that may increase their work fatigue (Boermans et al., 2013). In fact, fatigue is a systemic issue in military contexts (Goode, 2003; Gore et al., 2010) and is related to a range of undesirable outcomes, including cognitive impairments, reduced performance, and occupational injuries (Belenky et al., 2003; Nahrgang et al., 2011). Increasingly unrelenting military operational tempos have resulted in the redeployment of scarce resources between training and operations (Richard and Huffman, 2002), making fatigue an increasingly preoccupying phenomenon for military organizations (Caldwell and Caldwell, 2016). Moreover, although most military research has looked at fatigue and fatigue-related factors in deployed combat settings (see Miller et al., 2008; Rabinowitz et al., 2009; Gore et al., 2010), fatigue has also been identified as a pervasive phenomenon in the non-deployed military contexts – closer to the civilian context (Frone and Blais, 2019). Defense organizations rely on the safety, health, and performance of non-deployed personnel for operational readiness and resiliency (Department of National Defence and the Canadian Armed Forces, 2018; Frone and Blais, 2019) making it equally important to focus on fatigue in this context. These differences in job demands raise the possibility that military personnel would present higher levels of global and specific (mental, emotional, and physical) work fatigue when compared to civilians. In the present study, all the predictors and outcomes (described in the upcoming section) were only available in a Military sample. These unique characteristics of the Military context made it critical for us to verify whether and how the nature of the profiles would generalize to a more diversified set of civilian employees, or whether they would be specific to the Military context. However, lacking prior studies on work fatigue profiles, we leave open the question of whether and how profiles differ across military and civilian samples.

### Relationships Between Profiles and Covariates

A second critical step in the assessment of the construct validity of profiles is to document their theoretical and practical implications via the examination of associations between profile membership and theoretically-relevant covariates (Marsh et al., 2009; Meyer and Morin, 2016). Indeed, without information related to the key determinants of work fatigue profiles, or without the ability to document their consequences, simple knowledge regarding the nature of these profiles is likely to be of very limited utility for managers and organizations. In this study, we focus on the role of psychological empowerment in the prediction of profile membership, and on career satisfaction, job satisfaction, and turnover intentions as outcomes of profile membership.

#### Psychological Empowerment (PE)

Psychological empowerment is defined as a “set of psychological states that are necessary for an individual to feel a sense of control in relation to their work” (Spreitzer, 2008, p. 56). PE is a core psychological resource, allowing employees allowing to act in a volitional manner at work (Thomas and Velthouse, 1990; Spreitzer, 1995), and encompasses four work-related cognitions of competence, autonomy, impact, and meaning (Spreitzer, 2008). Competence refers to feelings of having the abilities required for a successful execution of their work, a cognition close to self-efficacy. Autonomy refers to feelings of being in control when initiating and regulating work behaviors. Impact refers to the perceived ability to influence operational, strategic, or administrative work outcomes. Finally, meaning occurs when employees feel that there is a good fit between work requirements and their own personal beliefs, standards, and values. Despite being distinct, these four cognitions have been shown to converge on a global PE construct (Seibert et al., 2011; Morin et al., 2016c).

Psychological empowerment value for organizations stems from its role as a psychological resource that can help workers handle the stressfulness of their work. Meta-analyses support the role of PE as a driver of organizationally-relevant outcomes, including turnover intentions, psychological health indicators, and in-role and extra-role performance (Seibert et al., 2011; Maynard et al., 2012; Maynard et al., 2013). Despite the important role of PE for a variety of individual and organizational outcomes, no studies have yet examined the role of this construct as a predictor of work fatigue (Frone and Tidwell, 2015). Nevertheless, prior studies have demonstrated the role of PE in the prediction of similar constructs, such as burnout. More precisely, Calvo and García (2018) have shown that meaning, competence, autonomy, and impact were negatively related to employees’ exhaustion levels. Livne and Rashkovits (2018) also reported a negative association between PE and burnout. In sum, these findings suggest that higher levels of PE should be associated with a higher probability of membership into profiles presenting lower global levels of work fatigue.

#### Outcomes

Finally, we also examine the associations between the work fatigue profiles and attitudinal outcome variables (job satisfaction, career satisfaction, and turnover intentions) previously shown to share variable-centered significant associations with work fatigue (Frone and Tidwell, 2015). Moreover, these attitudes were also considered given their documented positive effects on work performance (e.g., Bowling et al., 2015). For instance, employees’ levels of satisfaction with their job (e.g., Whitman et al., 2010) and career (e.g., Guan et al., 2017; Dubbelt et al., 2019) have both been found to share positive associations with work performance and persistence. Moreover, employees’ turnover intentions are known to represent the main predictor of voluntary turnover for a variety of organizations (Heavey et al., 2013; Rubenstein et al., 2018), an association that is particularly important in the military (Griffeth et al., 2000; Lytell and Drasgow, 2009). The cost of turnover intentions and turnover in terms of organizational performance and recruitment and training are also known to be immense (Heavey et al., 2013).

Although we expect well-differentiated associations between the work fatigue profiles and the outcome variables measured in the present research, the lack of previous person-centered studies of work fatigue profiles precludes the formulation of more specific hypotheses. In addition, previous person-centered studies of job burnout and psychological health and well-being did not systematically investigate the role of profile membership for the specific outcomes considered in the current research. In fact, the characteristics of the profiles identified in these previous studies (e.g., Leiter and Maslach, 2016) only allow us to hypothesize that profiles presenting lower levels of work fatigue should be characterized by higher career and job satisfaction, and lower turnover intentions relative to profiles presenting higher levels of work fatigue.

### The Current Research

This study relies on a methodological framework recently proposed by Morin et al. (2016b, 2017) in order to achieve a more comprehensive representation of the multidimensionality of work fatigue measurement and profiles. This study is thus globally relevant to research conducted within the field of applied psychology in providing an illustration of the application of this combined variable- and person-centered approach. More specifically, this study contributes to improve our understanding of work fatigue by: (a) better documenting the global and specific nature of this multidimensional construct; (b) achieving a clearer representation of the work fatigue configurations that best characterize employees’ individual profiles; and (c) investigating the generalizability of these profiles and their relations with PE and key outcome variables.

## Materials and Methods

### Participants

#### Military Sample

A stratified random sample of non-deployed Royal Canadian Air Force employees was identified from a sampling frame of 16,010 personnel. Samples were drawn randomly from six organizational strata using a proportional allocation based on sex, military status (civilians, Primary Reserve, and Regular Force), sex, years of service for the civilians, and rank for the military (non-commissioned members and officers) to ensure a proper representation on these characteristics. Sampling weights were created so that the sample more closely represented the target population.

The Canadian Armed Forces Social Science Research Review Board approved the data collection procedure. The online survey was accessible between May and August 2018. During this period, sampled employees were invited to complete the questionnaire using email or postcards. All participants were guaranteed that only aggregate data would be reported, and provided informed consent. This process resulted in 1,436 participants completing the questionnaire (for a response rate of 40%), including 83% of males. Thirty-three percent were younger than 35 years of age, 53% were between 35 and 54 years of age, and 14% were 55 years of age or older. Thirty-two percent of the participants had been with the organization for 1 to 10 years, 34% for 11 to 19 years, and 34% for 20 years or more.

#### Civilian Sample

Data were drawn from the random-digit dialed National Survey of Work Stress and Health. This survey sampled 2,975 adults working in the United States civilian labor force from December 2008 to May 2012 (for more details on the design, weights, and participant characteristics, see Frone and Tidwell, 2015, Study 2). Of all selected individuals, 47% completed the survey. For purposes of this study, we focus on the 2,477 wage and salary workers included in this sample (i.e., excluding owners/operators). Sampling weights were calculated so that the sample more closely represented the target population (Frone and Tidwell, 2015). Fifty-two percent of participants were male. Participants had an average tenure of 5 years and an average age of 40 years (Frone and Tidwell, 2015).

### Measures

#### Fatigue (Profile Indicators)

Participant’s levels of physical (α_*military*_ = 0.968; α_*civilian*_ = 0.937; e.g., *How often did you feel physically drained at the end of the workday?*), mental (α_*military*_ = 0.977; α_*civilian*_ = 0.949; e.g., *How often did you want to mentally shut down at the end of the workday?*), and emotional (α_*military*_ = 0.985; α_*civilian*_ = 0.954; e.g., *How often did want to avoid anything that took too much emotional energy at the end of the workday?*) fatigue were measured with the 18-item Three-Dimensional Work Fatigue Inventory (Frone and Tidwell, 2015). The time frame for the instructions was changed from the original past 12 months to the past month (*During the past month*…) to lessen recall bias. Items were rated on a five-point frequency response scale ranging from 1-*Everyday* to 5-*Never*. Items were reversed coded so that higher ratings represented higher levels of fatigue.

#### Psychological Empowerment (Predictor)

Participants’ perceptions of impact (three items; α = 0.917; e.g., *I have significant influence over what happens in my department*) and feelings of job meaningfulness (three items; α = 0.955; e.g., *The work I do is meaningful to me*) were assessed with the relevant subscales from Spreitzer’s (1995) questionnaire. Their perceptions of competence (four items; α = 0.895; e.g., *I am good at the things I do in my job*) and autonomy (six items; α = 0.805; e.g., *I feel free to do my job the way I think it could best be done*) at work were measured with the relevant subscales from Van den Broeck et al.’s (2010) questionnaire. Items were rated on a five-point response scale ranging from 1-*Totally Disagree* to 5-*Totally Agree*.

#### Turnover Intentions (Outcome)

Participants’ intentions to leave their occupation were assessed with Colarelli’s (1984) measure (three items; α = 0.800; e.g., *I frequently think of quitting my job*) using a five-point response scale (1-*Strongly Disagree* to 5-*Strongly Agree*).

#### Career Satisfaction (Outcome)

Participants’ career satisfaction was measured with three items (Peach, 2015; α = 0.790; e.g., *All things considered, how satisfied are you with the way your career is managed?*), rated using a seven-point scale (1-*Completely Dissatisfied* to 7-*Completely Satisfied*).

#### Job Satisfaction (Outcome)

Job satisfaction was assessed with one item (Peach, 2015; *Overall, I am satisfied with my job*), rated on a five-point scale (1-*Strongly Disagree* to 5-*Strongly Agree*).

### Analyses

#### Preliminary Analyses

Preliminary analyses were first conducted to verify the psychometric properties of all questionnaires and their measurement invariance across the military and civilian samples (Millsap, 2011). Factor scores were extracted from these analyses in standardized units (with a mean of 0 and a standard deviation of 1) and were used as profile indicators, predictors, and outcomes in the main analyses. The reliance on factor scores made it possible to achieve a partial control for unreliability (Skrondal and Laake, 2001) and to maintain the psychometric properties of the measurement models (i.e., bifactor, invariance; Morin et al., 2016b, 2017) while maximizing the simplicity of the estimated models (Morin et al., 2016d). Participants’ ratings of emotional, mental, and physical work fatigue were represented via a bifactor confirmatory factor analysis including one global factor (G-factor: Global fatigue) and three specific orthogonal factors (S-factors: Mental fatigue, emotional fatigue, and physical fatigue) (Morin et al., 2016b). This model was estimated separately for both samples (military: *n* = 1,436; civilian: *n* = 2,477) before being combined into a single model for tests of measurement invariance (Millsap, 2011). A bifactor approach (a) rely on the estimation of orthogonal factors (i.e., correlations among specific factors is assumed to be entirely explained by the global factor); (b) estimate a global factor reflecting the variance shared by all indicators; and (c) estimate specific factors reflecting the variance uniquely associated to each specific components. Bifactor models thus make it possible to consider the forest (i.e., global work fatigue) and the trees (i.e., emotional, physical, and mental work fatigue) in the subsequent analyses (Tóth-Király et al., 2018). This approach is consistent with the high correlations typically observed among fatigue components (Frone et al., 2018), and with research supporting a similar operationalization of burnout (Shirom and Melamed, 2006; Isoard-Gautheur et al., 2018). Importantly, this approach has been recommended to achieve the identification of clearer profiles in situations where a global construct (i.e., fatigue) co-exists with specificities measured from the same indicators (Morin et al., 2016b, 2017).

Because predictors and outcomes were only available for the military sample, a separate measurement model was estimated for these variables (with the exception of job satisfaction which was assessed using one item and standardized prior to its inclusion in the main analyses). This model included independent factors reflecting career satisfaction and turnover intentions. It also included a bifactor representation of psychological empowerment (PE), incorporating a global PE factor and orthogonal specific autonomy, meaning, impact, and competence factors. The decision to rely on a bifactor representation of PE is predicated on accumulated evidence that this construct encompasses co-existing global and specific dimensions (Seibert et al., 2011), as well as on the ability of bifactor models to separately assess the contribution of global and specific components in a way that remains untainted by multicollinearity (Morin et al., 2016a) and redundancy (Morin et al., 2016b, 2017). These preliminary analyses are disclosed in the online supplements. In particular, correlations among these factor scores and reliability information (ω = 0.726–0.995, *M* = 0.904) are reported in Supplementary Table 4 of these supplements.

#### Latent Profile Analyses and Test of Profile Similarity

Latent Profile Analyses (LPA) solutions including one to eight profiles were estimated separately in both samples (military and civilian), allowing the means and variances of the profile indicators (the fatigue factor scores) to differ across profiles (Peugh and Fan, 2013; Diallo et al., 2016). These analyses were used to determine if the same number of profiles would be identified across samples, before combining them into a multi-group solution for tests of profile similarity. Test of similarity were conducted in sequence (Morin et al., 2016d): (a) configural similarity (i.e., identical number of profiles); (b) structural similarity (i.e., identical within-profile means on the profile indicators); (c) dispersion similarity (i.e., identical within-profile variation on the profile indicators); and (d) distributional similarity (i.e., identical profile sizes). The parameter estimates from the most similar of these solutions were then retained for tests of associations between the profiles, their predictors, and outcomes. These additional tests were only conducted in the military sample, as the predictor and outcomes were not measured among the civilians.

#### Predictors and Outcomes

To incorporate predictors and outcomes to a single-sample solution fully equivalent to that estimated in the most similar multi-group solution, while ensuring that no change in profile definition occurs following the incorporation of predictors and outcomes (e.g., Diallo et al., 2017), we relied on the manual implementation of the three-step approach advocated by Morin and Litalien (2017; see also Asparouhov and Muthén, 2014). This approach allowed us to directly incorporate the predictors (using a multinomial logistic regression function) and outcomes (using the direct inclusion approach and the multivariate delta method: Raykov and Marcoulides, 2004) into the final solution.

#### Model Estimation and Selection

The main analyses were realized using the Maximum Likelihood Robust (MLR) estimator implemented in Mplus 8.3 (Muthén and Muthén, 2019), while incorporating stratified sampling weights (themselves calculated while taking into account participants nesting into work units) using Mplus complex survey design functionalities (Asparouhov, 2005). As these analyses relied on factor scores, no missing data had to be taken into account in these analyses. Indeed, the small amount of missing data at the item level (0–10.7%, *M* = 1.1%) was handled in the preliminary analyses reported in the online supplements and used to generate factor scores. To avoid local solutions, LPA were estimated with 10,000 randomly generated sets of starting values and 1,000 iterations; final optimization was conducted on the 500 best solutions (Hipp and Bauer, 2006).

For both samples, the final number of profiles was determined by considering the theoretical nature and meaning of the profiles, as well as by consulting statistical indicators to guide model selection (Muthén, 2003). The following indicators were used to guide model selection: (i) the Akaïke (1987) Information Criterion (AIC); (ii) the Consistent AIC (CAIC; Bozdogan, 1987); (iii) the Bayesian Information Criterion (BIC; Schwartz, 1978); (iv) the sample-size Adjusted BIC (ABIC; Sclove, 1987); and (v) the adjusted Lo et al. (2001) Likelihood Ratio Test (aLMR)^1^. Lower AIC, CAIC, BIC, and ABIC values indicate better fit, whereas a statistically significant aLMR value supports the value of the target solution when compared to a solution including one less profile.

The utility of the CAIC, BIC, ABIC, and BLRT has been well documented by statistical simulation studies, which have also called into question the appropriateness of the AIC and aLMR as guides for model selection (e.g., Nylund et al., 2007; Tofighi and Enders, 2008; Peugh and Fan, 2013; Tein et al., 2013; Diallo et al., 2016, 2017). As such, the latter indicators will be reported for purposes of ensuring a full disclosure of results, but not used for guiding model selection. Furthermore, the sample size dependency of these indicators can lead them to keep on supporting the addition of profiles beyond the optimal solution (Marsh et al., 2009). For this reason, it is recommended to rely on a graphical representation of these indicators (i.e., an elbow plot) to better locate the inflection point in the reduction of the value of the CAIC, BIC, and ABIC (Morin et al., 2011a). In tests of profile similarity, at least two indices out of the BIC, ABIC, and CAIC should be lower to support the more similar solution (Morin et al., 2016d). It should be noted that we also report the entropy, as a summary of the model classification accuracy (ranging from 0 to 1 with higher value reflecting greater accuracy). However, the entropy is a purely descriptive index that should not be used to guide model selection.

## Results

### Profiles and Profile Similarity

Results associated with all LPA solutions are reported in Table 1. Sample-specific elbow plots are presented in Supplementary Figures 1,2 of the online supplements. In the military sample, all indicators (CAIC, BIC, and ABIC) reach their lowest value at six profiles. For the civilian sample, the indicators fail to reach a clear minimum, and appear to decrease steadily at least until reaching the seven-profile solution. Looking closely at the solutions including five to eight profiles for both samples, it is interesting to note that additional profiles seem to present a meaningfully distinct shape, and thus to bring value to the solution, up to the six-profile solution (thus confirming the statistical information obtained in the military sample). Conversely adding a seventh or eighth profile simply led to the arbitrary division of existing profiles into similarly-shaped ones, and sometimes (i.e., eight-profile solution, military sample) in the estimation of empty profiles. Importantly, all solutions already displayed a very high level of correspondence across samples, thus providing early evidence of configural similarity. For all of those reasons, the six-profile solution was retained for both samples.

**TABLE 1:** Results from the latent profile analyses for each group and tests of profile similarity.

Model	LL	#fp	SC	AIC	CAIC	BIC	ABIC	Entropy	aLMR
**Latent profile analysis: military sample**									
1 Profile	−6177.466	8	1.825	12370.932	12421.089	12413.089	12387.675	Na	Na
2 Profiles	−5709.791	17	1.522	11453.582	11560.166	11543.166	11489.162	0.592	≤0.001
3 Profiles	−5522.327	26	1.766	11096.653	11259.663	11233.663	11151.070	0.734	0.022
4 Profiles	−5129.037	35	1.684	10328.075	10547.511	10512.511	10401.328	0.887	0.003
5 Profiles	−4988.225	44	1.808	10064.450	10340.313	10296.313	10156.540	0.853	0.172
6 Profiles	−4893.618	53	1.916	9893.237	10225.526	10172.526	10004.163	0.844	0.574
7 Profiles	−4975.300	62	1.682	10074.601	10463.317	10401.317	10204.364	0.803	0.389
8 Profiles	−4923.696	71	1.801	9989.393	10434.535	10363.535	10137.992	0.880	0.240
**Latent profile analysis: civilian sample**									
1 Class	−10885.798	8	2.584	21787.595	21842.114	21834.114	21808.696	Na	Na
2 Class	−9927.484	17	4.302	19888.967	20004.819	19987.819	19933.806	0.998	0.356
3 Class	−9693.761	26	3.854	19439.521	19616.706	19590.706	19508.098	0.965	0.381
4 Class	−9442.853	35	3.330	18955.705	19194.223	19159.223	19048.020	0.854	0.020
5 Class	−9237.109	44	3.716	18562.217	18862.069	18818.069	18678.270	0.766	0.495
6 Class	−9030.262	53	3.130	18166.524	18527.709	18474.709	18306.315	0.802	0.460
7 Class	−8849.489	62	3.173	17822.977	18245.495	18183.495	17986.506	0.811	0.567
8 Class	−8735.598	71	3.002	17613.196	18097.047	18026.047	17800.462	0.811	0.162
**Profile similarity tests: 6 profiles**									
Configural	−16763.837	107	2.557	33741.674	34519.785	34412.785	34072.787	0.826	
Structural	−16925.191	83	2.196	34016.382	34619.963	34536.963	34273.227	0.845	
Partial structural (5 profiles)	−16793.574	87	2.250	33761.149	34393.818	34306.818	34030.372	0.816	
Dispersion (5 profiles)	−16783.869	67	3.021	33701.737	34188.965	34121.965	33909.070	0.887	
Distributional	−16770.562	62	3.082	33665.123	34115.991	34053.991	33856.983	0.846	

*LL, loglikelihood; #fp, free parameters; SC, scaling correction; AIC, Akaïke information criterion; CAIC, consistent AIC; BIC, Bayesian information criterion; ABIC, sample-size adjusted BIC; aLMR, Lo-Mendel and Rubin’s likelihood ratio test; Na, not applicable.*

The results related to the tests of profile similarity, which can be consulted in the lowest part of Table 1, were thus conducted on this solution. These tests revealed an increase in the value of all indicators following the imposition of structural similarity to the model, suggesting structural differences across samples. We thus investigated partial structural similarity by relaxing equality constraints on the within-profile means in one out of the six profiles. This solution of partial structural similarity led to a decrease in all indicators compared to the configural similarity solution, and was thus supported by the data. Dispersion similarity was thus only imposed on the five structurally invariant profiles, and was supported by the data as shown by a reduction in the value of all indicators. Given that distributional similarity is an all-or-none characteristic across pairs of samples (Morin et al., 2016d), this last step involved imposing constraints on the size of all profiles (even the one that showed structural and dispersion differences). This model resulted in lower values on all indicators, and was thus supported. These results show that all profiles, even those differing across samples, can be considered to have the same relative size in both samples. This model was retained for interpretation and subsequent analyses.

**FIGURE 1:**
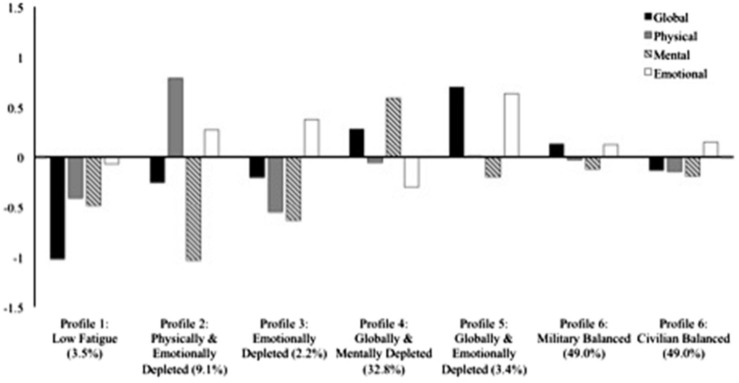
Final six-profile solution of distributional similarity. Profile indicators are factor scores with a mean of 0 and a standard deviation of 1.

This final model is illustrated in Figure 1, and parameter estimates are reported in Supplementary Table 5 of the online supplements. The first profile displays very low global levels of fatigue, low specific levels of physical and mental fatigue, and slightly below average specific levels of emotional fatigue. This *Low Fatigue* profile corresponds to 3.5% of the participants. The second profile is characterized by low global levels of fatigue, very high specific levels of physical fatigue, very low specific levels of mental fatigue, and high specific levels of emotional fatigue. This *Physically and Emotionally Depleted* profile corresponds to 9.1% of the participants. The third profile is characterized by low global levels of fatigue, very low specific levels of physical and mental fatigue, and high specific levels of emotional fatigue. This *Emotionally Depleted* profile is the smallest, and only corresponds to 2.2% of the participants. The fourth profile displays high global levels of fatigue, slightly below average specific levels of physical fatigue, very high specific levels of mental fatigue, and low specific levels of emotional fatigue. This *Globally and Mentally Depleted* profile corresponds to 32.8% of the participants. The fifth profile presents very high levels of global fatigue and specific emotional fatigue, average specific levels of physical fatigue, and slightly below average specific levels of mental fatigue. This *Globally and Emotionally Depleted* profile corresponds to 3.4% of participants. The sixth, and largest, profile corresponds to 49% of the participants. In the military sample, this profile presents slightly above average levels of global fatigue and specific emotional fatigue, and slightly below average specific levels of physical fatigue and mental fatigue. In the civilian sample this profile is characterized by slightly below average levels of global, specific physical, and specific mental fatigue, and by slightly above average specific levels of emotional fatigue. As such, whereas both samples displayed a globally *Balanced* profile where levels on all global and specific fatigue indicators remain close to the sample mean, the key difference between these two samples lies in the observation of fatigue levels that are very slightly higher in the military sample than in the civilian sample.

### PE and Profile Membership

The multinomial logistic regression results related to the role of PE in the prediction of profile membership are reported in Table 2. First, participants who reported higher global PE levels had a higher likelihood of belonging to the *Physically and Emotionally Depleted* (2) compared to the *Globally and Mentally Depleted* (4) and *Globally and Emotionally Depleted* (5) profiles. When considering the specific PE factors, it is first noteworthy that no association with profile membership was found for the PE meaning factor. The specific PE autonomy factor was the one with the most widespread associations with profile membership, being related to an increased likelihood of membership into the *Low Fatigue* (1) profile in comparison to the *Globally and Emotionally Depleted* (5) and *Balanced* (6) ones, as well as into the *Physically and Emotionally Depleted* (2), *Emotionally Depleted* (3) and *Globally and Mentally Depleted* (4) profiles relative to the *Globally and Emotionally Depleted* (5) one. Conversely, and unexpectedly, the specific PE impact factor was related to an increased likelihood of membership into the *Globally and Mentally Depleted* (4) profile in comparison to the *Physically and Emotionally Depleted* (2), *Emotionally Depleted* (3), and *Balanced* (6) profiles, as well as into the *Globally and Emotionally Depleted* (5) profile relative to the *Physically and Emotionally Depleted* (2) one. Likewise, specific PE competence levels predicted an increased likelihood of membership into the *Globally and Mentally Depleted* (4) profile relative to the *Emotionally Depleted* (3) one.

**TABLE 2:** Results from the multinomial logistic and multiple regressions predicting profile membership.

	**Profile 1 vs. Profile 6**		**Profile 2 vs. Profile 6**		**Profile 3 vs. Profile 6**		**Profile 4 vs. Profile 6**		**Profile 5 vs. Profile 6**	
					
**Predictors**	**Coeff (*SE*)**	**OR**	**Coeff (*SE*)**	**OR**	**Coeff (*SE*)**	**OR**	**Coeff (*SE*)**	**OR**	**Coeff (*SE*)**	**OR**
Global PE	0.436 (0.436)	1.547	0.393 (0.238)	1.481	0.089 (0.172)	1.093	−0.245 (0.137)	0.783	−0.189 (0.172)	0.828
Specific PE: autonomy	0.910 (0.464)*	2.484	0.389 (0.315)	1.476	0.190 (0.188)	1.209	0.178 (0.152)	1.195	−0.322 (0.178)	0.725
Specific PE: meaning	−0.122 (0.362)	0.885	−0.210 (0.422)	0.811	−0.260 (0.252)	0.771	0.142 (0.165)	1.153	0.122 (0.172)	1.130
Specific PE: impact	0.213 (0.332)	1.237	−0.411 (0.267)	0.663	−0.132 (0.175)	0.876	0.364 (0.144)*	1.439	0.240 (0.155)	1.271
Specific PE: competence	−0.165 (0.332)	0.848	−0.098 (0.245)	0.907	−0.302 (0.187)	0.739	0.088 (0.122)	1.092	−0.033 (0.171)	0.968

	**Profile 1 vs. Profile 5**		**Profile 2 vs. Profile 5**		**Profile 3 vs. Profile 5**		**Profile 4 vs. Profile 5**		**Profile 1 vs. Profile 4**	
					
**Predictors**	**Coeff (*SE*)**	**OR**	**Coeff (*SE*)**	**OR**	**Coeff (*SE*)**	**OR**	**Coeff (*SE*)**	**OR**	**Coeff (*SE*)**	**OR**

Global PE	0.625 (0.457)	1.868	0.582 (0.269)*	1.790	0.278 (0.221)	1.320	−0.056 (0.185)	0.946	0.681 (0.440)	1.976
Specific PE: autonomy	1.232 (0.483)*	3.428	0.711 (0.334)*	2.036	0.512 (0.231)*	1.669	0.500 (0.193)**	1.649	0.732 (0.464)	2.079
Specific PE: meaning	−0.244 (0.382)	0.783	−0.332 (0.425)	0.717	−0.382 (0.278)	0.682	0.020 (0.189)	1.020	−0.264 (0.367)	0.768
Specific PE: impact	−0.027 (0.351)	0.973	−0.651 (0.291)*	0.522	−0.372 (0.212)	0.689	0.124 (0.172)	1.132	−0.151 (0.339)	0.860
Specific PE: competence	−0.133 (0.365)	0.875	−0.065 (0.279)	0.937	−0.270 (0.236)	0.763	0.121 (0.183)	1.129	−0.254 (0.341)	0.776

	**Profile 2 vs. Profile 4**		**Profile 3 vs. Profile 4**		**Profile 1 vs. Profile 3**		**Profile 2 vs. Profile 3**		**Profile 1 vs. Profile 2**	
					
**Predictors**	**Coeff (*SE*)**	**OR**	**Coeff (*SE*)**	**OR**	**Coeff (*SE*)**	**OR**	**Coeff (*SE*)**	**OR**	**Coeff (*SE*)**	**OR**

Global PE	0.638 (0.250)*	1.893	0.334 (0.188)	1.397	0.347 (0.458)	1.415	0.304 (0.268)	1.355	0.043 (0.477)	1.044
Specific PE: autonomy	0.211 (0.327)	1.235	0.012 (0.200)	1.012	0.720 (0.483)	2.054	0.199 (0.331)	1.220	0.521 (0.538)	1.684
Specific PE: meaning	−0.352 (0.433)	0.703	−0.403 (0.267)	0.668	0.138 (0.423)	1.148	0.050 (0.455)	1.051	0.088 (0.533)	1.092
Specific PE: impact	−0.775 (0.294)**	0.461	−0.496 (0.193)*	0.609	0.345 (0.356)	1.412	−0.279 (0.287)	0.757	0.624 (0.395)	1.866
Specific PE: competence	−0.186 (0.261)	0.830	−0.391 (0.200)*	0.676	0.137 (0.370)	1.147	0.205 (0.284)	1.228	−0.068 (0.396)	0.934

**p < 0.01, *p < 0.05; SE, standard error of the coefficient; OR, odds ratio; the coefficients and OR reflect the effects of the predictors on the likelihood of membership into the first listed profile relative to the second listed profile; predictors are factor scores with a mean of 0 and a standard deviation of 1; PE: Psychological Empowerment; Profile 1: Low Fatigue; Profile 2: Physically and Emotionally Depleted; Profile 3: Emotionally Depleted; Profile 4: Globally and Mentally Depleted; Profile 5: Globally and Emotionally Depleted; Profile 6: Balanced.

### Profile Membership and Outcomes

**FIGURE 2:**
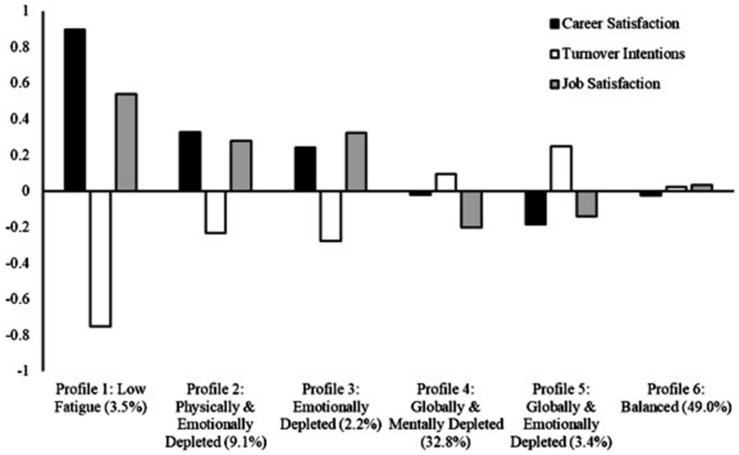
Outcome means for the six-profile distributional similarity model. Outcomes are factor scores with a mean of 0 and a standard deviation of 1.

Relations between profiles and outcomes are reported in Table 3, and illustrated in Figure 2. The highest levels of career satisfaction were observed in the *Low Fatigue* (1) profile, followed by the *Physically and Emotionally Depleted* (2) and *Emotionally Depleted* (3) profiles, which did not differ from one another, and then by the *Globally and Mentally Depleted* (4), *Globally and Emotionally Depleted* (5), and *Balanced* (6) profiles, which did not differ from one another. The highest levels of turnover intentions were observed in the *Globally and Mentally Depleted* (4) and *Globally and Emotionally Depleted* (5) profiles, followed by the *Low Fatigue* (1), *Physically and Emotionally Depleted* (2), and *Emotionally Depleted* (3) profiles, which did not differ from one another. Turnover intentions were also more pronounced in the *Globally and Emotionally Depleted* (5) profile than in the *Balanced* (6) profile, and more pronounced in this *Balanced* (6) profile than in the *Low Fatigue* (1) and *Emotionally Depleted* (3) profiles. However, turnover intentions observed in the *Balanced* (6) profile did not differ from those observed in the *Physically and Emotionally Depleted* (2) and *Globally and Mentally Depleted* (4) profiles. Finally, the highest levels of job satisfaction were observed in the *Low Fatigue* (1), *Physically and Emotionally Depleted* (2), and *Emotionally Depleted* (3) profiles, which did not differ from one another, followed by the *Globally and Mentally Depleted* (4) and *Globally and Emotionally Depleted* (5) profiles, which also did not differ from one another. Levels of job satisfaction were also higher in the *Low Fatigue* (1) and *Emotionally Depleted* (3) profiles than in the *Balanced* (6) profile, and in this *Balanced* (6) profile than in the *Globally and Mentally Depleted* (4) profile. However, job satisfaction levels in the *Balanced* (6) profile did not differ from those observed in the *Physically and Emotionally Depleted* (2) and *Globally and Emotionally Depleted* (5) profiles.

**TABLE 3:** Associations between profile membership and the outcomes.

	Profile 1	Profile 2	Profile 3	Profile 4	Profile 5	Profile 6	Summary of significant
	Mean [CI]	Mean [CI]	Mean [CI]	Mean [CI]	Mean [CI]	Mean [CI]	differences
Career satisfaction	0.897	0.326	0.240	−0.022	−0.184	−0.026	1 > 2 = 3 > 4 = 5 = 6
	[0.481; 1.313]	[0.063;0.589]	[0.015;0.465]	[−0.142;0.1098]	[−0.356; −0.012]	[−0.122;0.070]	
Turnover intentions	−0.750	−0.232	−0.276	0.095	0.249	0.023	4 = 5 > 1 = 2 = 3; 5 > 6 > 1 = 3; 2 = 6; 4 = 6
	[−0.750; −0.311]	[−0.516;0.052]	[−0.509; −0.043]	[−0.028;0.218]	[0.063;0.435]	[−0.071;0.117]	
Job satisfaction	0.539	0.280	0.323	−0.203	−0.139	0.033	1 = 2 = 3 > 4 = 5; 1 = 3 > 6 > 4; 2 = 6; 5 = 6
	[0.057; 1.021]	[−0.063;0.623]	[0.082;0.564]	[−0.350; −0.056]	[−0.355;0.077]	[−0.075;0.141]	

*M, mean; [CI]: 95% confidence interval; outcomes are factor scores with a mean of 0 and a standard deviation of 1; Profile 1: low fatigue; Profile 2: physically and emotionally depleted; Profile 3: emotionally depleted; Profile 4: globally and mentally depleted; Profile 5: globally and emotionally depleted; Profile 6: balanced.*

## Discussion

This study sought to document work fatigue profiles, and to do so while being able to rely on a representation of work fatigue allowing us to better understand the globality and specificity of this multidimensional construct. Importantly, this study also documented the construct validity and meaningfulness of the extracted profiles by verifying the extent to which they would be replicated across samples of military and civilian employees, by investigating the effects of PE on profile membership, and by examining the effects of these profiles on attitudinal outcomes.

### Employees’ Work Fatigue Profiles

Despite some minor differences related to the shape of one of the profiles, which we will address shortly, six profiles best summarized the work fatigue configurations observed across samples of military and civilian employees: (1) *Low Fatigue*; (2) *Physically and Emotionally Depleted*; (3) *Emotionally Depleted*; (4) *Globally and Mentally Depleted*; (5) *Globally and Emotionally Depleted*; and (6) *Balanced*. These profiles matched our expectations, as well as results from person-centered studies of conceptually-related constructs (e.g., Leiter and Maslach, 2016; Morin et al., 2016b, 2017; Berjot et al., 2017). This similarity highlights the robustness of our results and the likely utility of interventions focused on specific profiles of employees.

These profiles reinforce the value of jointly considering global and specific (mental, emotional, and physical) facets of work fatigue. More precisely, our findings revealed that employees with globally low (*Low Fatigue*, *Physically and Emotionally Depleted*, and *Emotionally Depleted*) or high (*Globally and Mentally Depleted*, and *Globally and Emotionally Depleted*) levels of work fatigue displayed more imbalanced profiles where levels obtained on specific work fatigue dimensions tended to show some deviation from that global level and from the sample average. Conversely, employees characterized by a *Balanced* profile displayed a more equilibrated configuration (i.e., close to average and well-aligned levels of global and specific work fatigue). In particular, the identification of such a large (49.0%) *Balanced* profile suggests that global levels of work fatigue remain adequate and aligned across dimensions for almost half of the participants. This finding is aligned with results from past studies of work engagement (Gillet et al., 2019a), well-being and psychological health (Morin et al., 2016b, 2017) or need satisfaction (Gillet et al., 2019b), in which a similarly balanced profile was also found to characterized a large proportion of the sample.

However, it is noteworthy that in the military sample, the *Balanced* profile was found to be characterized by slightly above average levels of global work fatigue and specific emotional fatigue, and by slightly below average specific levels of physical and mental fatigue. In contrast, in the civilian sample, this *Balanced* profile was characterized by slightly below average levels of global, specific physical, and specific mental fatigue, and by slightly above average specific levels of emotional fatigue. In summary, the key difference between these two samples lies in the observation of slightly higher fatigue levels within this large, *Balanced*, normative profile among military employees relative to civilian employees. These findings are aligned with prior research suggesting that military personnel tend to present higher levels of work fatigue due to their constant exposition to a range of stressful conditions (Gore et al., 2010; Boermans et al., 2013).

### PE as a Determinant of Work Fatigue Profiles

Our findings showed widespread relations between PE and profile membership, thus supporting the role of PE as a key psychological resource involved in the prediction of a variety of desirable outcomes for employees (e.g., Seibert et al., 2011). More specifically, global PE levels and specific levels of PE autonomy both predicted a decreased likelihood of membership into least desirable profiles (i.e., those with higher global levels of work fatigue) and an higher likelihood of membership into the most desirable ones (i.e., with lower global levels of work fatigue). This finding is in line with variable-centered research showing that PE and autonomy are negatively related to burnout (Calvo and García, 2018; Livne and Rashkovits, 2018) and fatigue (Gillet et al., 2019b; Ybema et al., 2019).

Our results also show that specific levels of competence or impact that are not backed up or supported by sufficient levels of autonomy and meaning carry risks in terms of work fatigue. It is interesting to note that Gillet et al. (2019c) similarly showed, in a study of need satisfaction, that imbalance in the degree to which employees’ needs for competence were met relative to their needs for relatedness and autonomy were related to lower levels of helping behaviors. Employees with high levels of competence or feeling able to have an impact at work tend to persevere when faced with difficulties and may display perfectionist behaviors known to be positively related to burnout (Hill and Curran, 2016), which could possibly explain their higher levels of fatigue, especially when these employees feel restricted in their ability to enact their true potential (lack of autonomy), or when these efforts involve a job perceived as lacking in personal signification (lack of meaning). Other investigations should be conducted to replicate the present findings, and to examine the mechanisms at play in these unexpected relations.

Finally, imbalance in specific levels of PE meaning was not related to profile membership. This finding is different from what could be expected from previous variable-centered research (e.g., Hochwälder and Brucefors, 2005; Boudrias et al., 2012), which could be explained by the multivariate approach adopted in the present study in which PE facets were simultaneously considered via a bifactor representation. This approach made it possible for us to identify the most important determinants of profile membership, once the covariance between PE facets was considered. Indeed, as shown in Supplementary Table 4 of the online supplements, these facets were moderately correlated with one another and displayed similar univariate relations with the work fatigue dimensions when considered in isolation. These results encourage scholars to examine whether PE facets (Morin et al., 2016c) uniquely contribute to employees’ work fatigue profiles.

### Outcomes of Profile Membership

Our results finally revealed well-differentiated patterns of association between the work fatigue profiles and the different attitudinal outcomes assessed in the present study. These results are consistent with our hypotheses and with prior results (e.g., Frone and Tidwell, 2015; Leiter and Maslach, 2016), in supporting the effects of employees’ global levels of work fatigue on various outcomes. Indeed, despite a few differences, employees with low levels of global work fatigue (*Low Fatigue*, *Physically and Emotionally Depleted*, and *Emotionally Depleted*) were subjected to a more adaptive functioning (i.e., higher levels of job and career satisfaction, and lower levels of turnover intentions) than those presenting high global levels of work fatigue (*Globally and Mentally Depleted* and *Globally and Emotionally Depleted*). These findings are consistent with previous studies revealing that workers presenting high levels of work fatigue tend to experience tiredness and reduced functional capacity, which in turn increase their likelihood of experiencing undesirable outcomes (e.g., Frone and Tidwell, 2015).

However, not all profiles were found to systematically differ from one another on all outcomes in a way that matched our expectations (Frone and Blais, 2019). For instance, the levels of turnover intentions and job satisfaction observed in the *Low Fatigue*, *Physically and Emotionally Depleted*, and *Emotionally Depleted* profiles were impossible to differentiate. However, the *Low Fatigue* profile still displayed higher levels of career satisfaction than the other two, supporting the benefits of displaying low levels of work fatigue across dimensions.

Our results also suggest that exposure to misaligned levels of physical work fatigue, may be as problematic for turnover intentions and job dissatisfaction as exposure to globally moderate levels of work fatigue. There thus seems to be limits to the possible benefits of displaying a *Physically and Emotionally Depleted* profile given the high misaligned levels of physical work fatigue characteristic of this profile. However, the *Physically and Emotionally Depleted* and *Emotionally Depleted* profiles presented similar levels of job and career satisfaction and turnover intentions. The main distinction between these two profiles lies in the observation of high levels of physical work fatigue in the *Physically and Emotionally Depleted* profile and of low levels of physical work fatigue in the *Emotionally Depleted* profile, thus suggesting that the harmful effects of misaligned levels of physical work fatigue may be limited to specific outcomes.

When considered together our results thus reinforce, on the one hand, the idea that presenting more aligned or low levels of work fatigue tend to yield similar benefits in terms of turnover intentions and job satisfaction. As documented in the self-determination theory literature (e.g., Gillet et al., 2019b), our results showed that workers with a balanced level of work fatigue displayed similar levels of turnover intentions and job satisfaction than their colleagues displaying lower global levels of work fatigue as part of an unbalanced profile. Therefore, balance across work fatigue facets may stem from a more thoughtful allocation of work resources, which may in turn limit work-related stress and conflicts, thus leading to more adaptive consequences. On the other hand, our results also revealed some negative outcomes to be associated with the *Balanced* profile (e.g., low levels of career satisfaction), thus alluding to some limitation to the benefits of having a more balanced work fatigue configuration when compared to lower, yet more imbalanced, levels. More generally, our results confirm the utility of taking into account both global and specific facets of work fatigue when studying the implications of work fatigue profiles, as well as the value of considering a variety of outcomes. Additional studies considering a broader range of positive (e.g., job performance, organizational citizenship behaviors) and negative (e.g., absenteeism, work-family conflict) outcomes are needed to better understand the mechanisms underlying these relations.

### Practical Implications

From an intervention perspective, our findings demonstrate that supervisors should be particularly attentive to employees presenting low levels of PE, or to competent workers with a possibly strong impact who lack a sufficient level of freedom or job meaning perceptions. Indeed, our results showed that these workers were more likely to experience higher global levels of work fatigue, in turn leading them to experience lower levels of job and career satisfaction. Consequently, changes designed to promote employees’ balanced PE levels sustainably might decrease work fatigue in the long run. For instance, moving toward or enhancing high-involvement managerial systems (e.g., performance-related remuneration schemes) may help practitioners to improve employees’ PE (Rehman et al., 2019). Organizations would also benefit from interventions seeking to promote self-directed decision making, enactive mastery experiences, and opportunities for personal growth. Moreover, organizational changes seeking to promote task variety, significance, and identity might increase employees’ PE levels while helping to ensure balance among PE facets (Simonet et al., 2019). In particular, increases in task variety are likely to be most useful for employees who already have access to training opportunities in order to develop the skills required to tackle the new tasks, and to all relevant information (Parker, 1998). In situations where increases in task variety, significance or identity are not possible, interventions focused on task sequencing (Derfler-Rozin et al., 2016), or seeking to limit work overload while improving justice (Emery et al., 2019) might be viable alternatives.

In terms of research implications, the results obtained in this study showcase the importance of adequately taking into account the dual global and specific nature of work fatigue ratings. Indeed, failure to account for this form of multidimensionality is likely to mistakenly suggest that the mental, emotional, and physical facets of work are fatigue are reasonably distinct constructs without a common core and yet displaying comparable associations with covariates (Morin et al., 2016b, 2017). This erroneous conclusion would in fact stem from the unmodelled role played by workers’ global work fatigue levels, and serve to obscure the equally important role played by specific levels of imbalance associated with each fatigue component. Ignoring this duality will thus result in a biased, and far more limited, view of the complex reality of the work fatigue construct.

In terms of psychological assessment, our results indicate that a bifactor approach is required to avoid obtaining work fatigue estimates capturing a confusing blend of variance attributed to global and specific components likely to be contaminated by multicollinearity. This conclusion reinforces the value of latent variable methods. However, although latent variable methodologies are straightforward to apply in a research context, these approaches do not naturally lend themselves to the requirements of practitioners who want to obtain manifest scores on work fatigue measures. For such purposes, scoring procedures will need to be developed using calculations similar to those used to generate factor scores. Importantly, using the results from this study to fix parameter values, Mplus can be used in this manner (Perreira et al., 2018). Scores obtained using this approach will be naturally standardized and easy to interpret in relation to the sample means and variances, at least pending the formal development of more representative interpretative norms.

### Limitations and Future Directions

Although the present research offers the first investigation of the characteristics, determinants, and consequences of employees’ work fatigue profiles, it has some limitations. First, this study capitalized on self-report measures, which may have been influenced by self-reported biases and social desirability. Upcoming studies should incorporate more objective indicators of organizational and individual functioning (e.g., absenteeism), as well as ratings obtained from multiple informants (e.g., supervisors’ ratings of performance). Second, this study involved two samples of military and civilian employees, and predictors and outcomes were only available in the Military sample, calling into question the generalizability of these results. Other person-centered studies are needed to confirm the generalizability of the profiles identified here and their relations with a broader range of determinants and consequences across a variety of countries, cultures, and occupational groups (e.g., nurses, sales employees, and managers) (Morin et al., 2016d).

Third, we examined covariates specified as predictors (PE) or outcomes (job and career satisfaction, and turnover intentions) based on theoretical and empirical considerations (e.g., Frone and Tidwell, 2015). Although our approach made it possible to rule out possible effects of predictors on profile membership, our study design and the limitations inherent to our analytical method did not allow us to assess possible spurious associations, reversed causality, or reciprocal influence, nor the eventuality of profile membership impacting variations in outcome levels. Consequently, additional longitudinal research would gain from studying the direction of the relations between determinants, consequences, and profiles. In addition, longitudinal research would make it possible to confirm that the work fatigue profiles identified here are similar in terms of number, size, characteristics, variability, and consequences over time, and to test whether membership into these various profiles remains stable over time. Finally, we only considered a single predictor of profile membership (PE). It would be worthwhile for future studies to consider a greater variety of work-related (e.g., managerial behaviors) or individual (e.g., perfectionism, job crafting) predictors.

## Data Availability Statement

The data generated for this study is subject to the following licenses/restrictions: Security and confidentiality. Requests to access these datasets should be directed to A-RB, arblais@gmail.com.

## Ethics Statement

The studies involving human participants were reviewed and approved by the Canadian Armed Forces Social Science Research Review Board. The patients/participants provided their electronic informed consent to participate in this study.

## Author Contributions

All authors listed have made a substantial, direct and intellectual contribution to the work, and approved it for publication.

## Conflict of Interest

The authors declare that the research was conducted in the absence of any commercial or financial relationships that could be construed as a potential conflict of interest.

## Footnotes

1 One additional indicator, the Bootstrap Likelihood Ratio Test (BLRT), is not currently available in Mplus when using stratified sampling weights.
