# Supplementary material for: Work Fatigue Profiles: Nature, Implications, and Associations With Psychological Empowerment
Source: Front Psychol. 2020 Nov 26;11:596206. doi: 10.3389/fpsyg.2020.596206 (PMC7732697; doi:10.3389/fpsyg.2020.596206)
Supplement: Supplementary file 1 [file Data_Sheet_1.pdf]

Preliminary Analyses

Analytical Strategy

All preliminary measurement models were estimated using the robust weighted least square estimator with mean and variance adjusted statistics (WLSMV) implemented in Mplus 8.3 (Muthén & Muthén, 2019). This estimator is best suited to the ordinal nature of the Likert-type scales and asymmetric response thresholds of the instruments used in the present study (Bandalos, 2014; Beauducel & Herzberg, 2006; Finney & DiStephano, 2013; Flora & Curran, 2006; Lubke & Muthén, 2004; Rhemtulla, Brosseau-Liard, & Savalei, 2012). The limited amount of missing data present at the item level was handled using the full available information (Enders, 2010) via algorithms implemented in Mplus for WLSMV estimation (Asparouhov & Muthén, 2010). Goodness-of-fit was assessed using the WLSMV chi-square test of exact fit (χ^2), the comparative fit index (CFI), the Tucker-Lewis index (TLI), and the root mean square error of approximation (RMSEA) and its confidence intervals (Hu & Bentler, 1999; Marsh, Hau, & Grayson, 2005). However, only the sample-size independent goodness-of-fit indices (CFI, TLI, RMSEA) were used to empirically assess model fit (Marsh et al., 2005). Due to the known oversensitivity of the χ^2 to sample size and minor sources of misfit (Marsh et al., 2005), we considered that CFI and TLI values greater than .90 and .95, and RMSEA values lower than .08 and .06, respectively reflect adequate and excellent model fit (Hu & Bentler, 1999; Yu, 2002). In tests of measurement invariance conducted on the work fatigue questionnaire (Millsap, 2011) across the military and civilian samples, we followed guidelines proposed by Chen (2007) and Cheung & Rensvold (2002) suggesting that a decrease in CFI and TLI greater than .01, or an increase in RMSEA of more than .015, indicate that measurement invariance is not supported.

As noted in the main manuscript, work fatigue was operationalized using a bifactor confirmatory factor analytic (bifactor-CFA) measurement model (Morin et al., 2016). More precisely, this model included one global factor (G-factor: Global fatigue) and three specific orthogonal factors (S-factors: Mental, emotional, and physical fatigue), all set to be orthogonal according to bifactor assumptions

(Morin et al., 2016). This model also included 18 a priori correlated uniqueness to account for the identical wording of items across fatigue dimensions (Marsh et al., 2013). For purposes of comparison, we also assessed a simple CFA model including only the three a priori fatigue factors (physical, emotional, and mental). The fatigue model was first estimated separately for both groups, before being combined into a single model for tests of measurement invariance (Millsap, 2011). These tests were conducted in the following sequence, as adapted to WLSMV estimation (Millsap, 2011; Morin et al., 2016): (i) configural invariance (same model), (ii) weak invariance (identical factor loadings), (iii) strong invariance (identical response thresholds), (iv) strict invariance (identical item uniquenesses), (v) invariance of the correlated uniquenesses, (vi) invariance of the latent variance-covariance matrix, and (vii) invariance of the latent means.

For predictors and outcomes, a separate model was estimated in the military sample including two CFA factors reflecting career satisfaction and turnover intentions, and a bifactor operationalization of psychological empowerment (global, autonomy, meaning, impact, and competence; Seibert, Wang, & Courtright, 2011). This model included an a priori orthogonal method factor to control for the methodological artifact created by four negatively worded items (Zhang, Noor, & Savalei, 2016).

Results

The goodness-of-fit indices of all preliminary measurement models are reported in Table S1. These results support the adequacy of our a priori measurement models for fatigue in both samples, as well as our a priori measurement model for the predictors and outcomes in the military sample. For the work fatigue questionnaire, it is interesting to note that the CFA and bifactor-CFA measurement models achieved a very similar level of fit in both samples. However, although both of these models also resulted in satisfactory parameter estimates in terms of factor definition, the CFA model resulted in estimates of factor correlations among the fatigue dimensions that were high enough to suggest multicollinearity and conceptual redundancy (r = .602 to .870, M = .754). These CFA factor correlations thus support our

decision to rely on a methodological approach making it possible to disaggregate participants' global levels of fatigue from their specific levels of physical, emotional, and mental fatigue.

The results further supported the configural, weak, strong, and strict invariance of the retained bifactor-CFA solution across samples, as well as the invariance of the a-priori correlated uniquenesses. However, the results failed to support the complete invariance of the latent variance-covariance matrix, as shown by an increase in the RMSEA value greater than .015, leading us to pursue a solution of partial invariance. Investigation of the parameter estimates from the last supported solution (invariant correlated uniquenesses) and of the modification indices of the failed model (latent variance-covariance invariance) suggested that the global fatigue factor seemed to have a greater level of variability in the military sample relative to the civilian sample. As such, a model of partial invariance of the latent variance-covariance matrix was estimated, allowing the latent variance of this global factor to differ across groups, but keeping the equality constraints on the variances of the specific factor. Starting from this model, the results also supported the invariance of the latent means across samples. Factor scores were saved from this final model, resulting in profiles indicators that can be interpreted in standard deviations units from the sample mean (M = 0, SD = 1), with the sole exception of the global fatigue factor in the civilian sample which had a slightly lower level of variability (M = 0, SD = .588; Meyer & Morin, 2016).

The standardized parameter estimates from this final model are reported in Tables S2, together with composite reliability coefficients (ω; McDonald, 1970; Morin, Myers, & Lee, 2019). In the interpretation of bifactor results, it is important to keep in mind that bifactor models lead to a division of the true score (i.e., reliable) variance present at the item level into two (global and specific) factors so that factor loadings and reliability estimates are typically smaller (Morin et al., 2019). Likewise, it is frequent in bifactor solutions to observe that some items present a dominant association with one of these two factors, which simply indicate that these items are better indicators of one of these two layers of measurement (Morin et al., 2019). With this in mind, all global and specific factors were generally well-defined, and even more weakly defined factors still proved to be reliable for the military sample: (a) global fatigue (λ =

.766 to .937; *M* = .881; ω = .995); (b) physical fatigue (.380 to .532; *M* = .460; ω = .924); (c) mental fatigue (.190 to .341; *M* = .271; ω = .864); and (d) emotional fatigue (.362 to .420; *M* = .380; ω = .940). Similar conclusions apply to the civilian sample: (a) global fatigue (.573 to .844; *M* = .744; ω = .985); (b) physical fatigue (.528 to .705; *M* = .616; ω = .924); (c) mental fatigue (.290 to .510; *M* = .407; ω = .863); and (d) emotional fatigue (.521 to .597; *M* = .550; ω = .940).

Finally, the predictors and outcomes measurement model was also able to achieve a satisfactory level of model fit in the military sample and also resulted in satisfactory parameter estimates, reported in Table S3: (a) global empowerment (.232 to .813; *M* = .522; ω = .940); (b) specific autonomy (.158 to .676; *M* = .433; ω = .701); (c) specific meaning (.533 to .627; *M* = .576; ω = .912); (d) specific impact (.566 to .758; *M* = .689; ω = .888); (e) specific competence (.776 to .880; *M* = .834; ω = .939); (f) turnover intentions (.749 to .911; *M* = .824; ω = .866); and (g) career satisfaction (.769 to .773; *M* = .808; ω = .850).

References

Asparouhov, T., & Muthén, B.O. (2010). *Weighted Least Square estimation with missing data*. Los Angeles, CA: Muthén & Muthén.

Bandalos, D.L. (2014). Relative performance of categorical diagonally weighted least squares and robust maximum likelihood estimation. *Structural Equation Modeling*, *21*, 102-116.

Beauducel, A., & Herzberg, P. (2006). On the performance of Maximum Likelihood versus means and variance adjusted Weighted Least Squares estimation. *Structural Equation Modeling, 13*, 186-203.

Chen, F.F. (2007). Sensitivity of goodness of fit indexes to lack of measurement. *Structural Equation Modeling*, *14*, 464-504.

Cheung, G.W., & Rensvold, R.B. (2002). Evaluating goodness-of fit indexes for testing measurement invariance. *Structural Equation Modeling*, *9*, 233-255.

Enders, C.K. (2010). *Applied missing data analysis*. New York, NY: Guilford.

Finney, S.J., & DiStefano, C. (2013). Non-normal and categorical data in structural equation modeling. In G.R. Hancock & R.O. Mueller (Eds), *Structural equation modeling: A second course*, 2nd *edition* (pp.

439-492). Greenwich, CO: IAP.

Flora, D.B. & Curran, P.J. (2006). An empirical evaluation of alternative methods of estimation for confirmatory factor analysis with ordinal data. *Psychological Methods*, *9,* 466-491.

Hu, L.-T., & Bentler, P.M. (1999). Cutoff criteria for fit indexes in covariance structure analysis: Conventional criteria versus new alternatives. *Structural Equation Modeling, 6,* 1-55.

Lubke, G., & Muthén, B. (2004). Applying multigroup confirmatory factor models for continuous outcomes to Likert scale data complicates meaningful group comparisons. *Structural Equation Modeling*, *11*, 514-534.

Marsh, H.W., Abduljabbar, A.S., Abu-Hilal, M.M., Morin, A.J.S., Abdelfattah, F., Leung, K.C., Xu, M.K., Nagengast, B., & Parker, P. (2013). Factorial, convergent, and discriminant validity of TIMSS math and science motivation measures: A comparison of Arab and Anglo-Saxon countries. *Journal of Educational Psychology*, *105*, 108-128.

Marsh, H.W., Hau, K.-T., & Grayson, D. (2005). Goodness of fit in structural equation models. In A. Maydeu-Olivares & J.J. McArdle (Eds.), *Contemporary psychometrics* (pp. 275-340). Mahwah, NJ: Erlbaum.

McDonald, R.P. (1970). Theoretical foundations of principal factor analysis and alpha factor analysis. *British Journal of Mathematical and Statistical Psychology, 23*, 1-21.

Meyer, J.P., & Morin, A.J.S. (2016). A person-centered approach to commitment research: Theory, research, and methodology. *Journal of Organizational Behavior, 36*, 584-612.

Millsap, R.E. (2011). *Statistical approaches to measurement invariance*. New York, NY: Taylor & Francis.

Morin, A.J.S., Arens, A.K., Tran, A., & Caci, H. (2016). Exploring sources of construct-relevant multidimensionality in psychiatric measurement: A tutorial and illustration using the Composite Scale of Morningness. *International Journal of Methods in Psychiatric Research, 25,* 277-288.

Morin, A.J.S., Myers, N.D., & Lee, S. (2019, in press). Modern factor analytic techniques: Bifactor

models, exploratory structural equation modeling (ESEM) and bifactor-ESEM. In G. Tenenbaum & R.C. Eklund (Eds.), *Handbook of sport psychology, 4th Edition*. London, UK: Wiley

Muthén, L., & Muthén, B. (2019). *Mplus user's guide*. Los Angeles, CA: Muthén & Muthén.

Rhemtulla, M., Brosseau-Liard, P.É., & Savalei, V. (2012). When can categorical variables be treated as continuous? A comparison of robust continuous and categorical SEM estimation methods under suboptimal conditions. *Psychological Methods, 17*, 354-373.

Seibert, S.E., Wang, G., & Courtright, S.H. (2011). Antecedents and consequences of psychological and team empowerment in organizations: A meta-analytic review. *Journal of Applied Psychology, 96*, 981-1003.

Yu, C.Y. (2002). *Evaluating cutoff criteria of model fit indices for latent variable models with binary and continuous outcomes*. Los Angeles, CA: University of California.

Zhang, X., Noor, R., & Savalei, V. (2016). Examining the effect of reverse worded items on the factor structure of the seed for cognition scale. *PLoS ONE*, *11*, e0157795.

Table S1

Preliminary Measurement Models

Model	df	χ^2	CFI	TLI	RMSEA	RMSEA 90% CI	$\Delta\chi^2$ (Δdf)	ΔCFI	ΔTLI	ΔRMSEA
**Work Fatigue (Bifactor: Global, Mental, Physical, Emotional)**										
CFA (Military)	111	898.972*	.998	.997	.069	.065; .073				
CFA (Civilian)	111	965.094*	.993	.991	.055	.052; .058				
Bifactor-CFA (Military)	99	727.406*	.998	.997	.066	.062; .071				
Bifactor-CFA (Civilian)	99	662.542*	.995	.993	.048	.045; .051				
Configural invariance	198	1346.313*	.998	.996	.054	.052; .057				
Weak invariance	230	1302.205*	.998	.997	.049	.046; .051	167.151 (32)*	.000	+.001	-.005
Strong invariance	280	1391.741*	.998	.998	.045	.043; .047	261.461 (50)*	.000	+.001	-.004
Strict invariance	298	1957.691*	.997	.997	.053	.051; .056	438.778 (18)*	-.001	-.001	.008
Invariance of the correlated uniquenesses	316	1971.653*	.997	.997	.052	.050; .054	88.751 (18)*	.000	.000	-.001
Latent variance-covariance invariance	320	3801.041*	.993	.993	.075	.072; .077	369.237 (4)*	-.004	-.004	+.023
Partial latent variance-covariance invariance	319	1728.641*	.997	.997	.048	.045; .050	22.589 (3)*	.000	.000	-.004
Latent means invariance	323	1484.202*	.998	.998	.043	.041; .045	48.205 (4)*	+.001	+.001	-.005
**Predictors and Outcomes**	179	1019.028*	.986	.982	.057	.054; .061				

Note. **p* < .01; df: Degrees of freedom; CFA: Confirmatory factor analysis; χ^2: Chi-square; CFI: Comparative fit index; TLI: Tucker-Lewis index; RMSEA: Root mean square approximation; CI: 90% confidence intervals for the RMSEA; $\Delta\chi^2$: Chi-square difference test.

Table S2

Standardized Parameter Estimates for the Final Work Fatigue Measurement Model (Latent Means Invariance with Partial Variance-Covariance Invariance)

	Military Sample					Civilian Sample				
	Global Fatigue (λ)	Physical (λ)	Mental (λ)	Emotional (λ)	δ	Global Fatigue (λ)	Physical (λ)	Mental (λ)	Emotional (λ)	δ
Item 1	.766	.471			.130	.573	.611			.212
Item 2	.788	.532			.158	.601	.678			.266
Item 3	.810	.532			.060	.631	.705			.105
Item 4	.868	.383			.100	.716	.538			.198
Item 5	.853	.459			.061	.693	.635			.116
Item 6	.858	.380			.120	.700	.528			.231
Item 7	.905		.305		.088	.781		.448		.189
Item 8	.923		.259		.082	.815		.389		.184
Item 9	.919		.341		.039	.808		.510		.087
Item 10	.937		.215		.076	.844		.330		.179
Item 11	.928		.313		.041	.826		.473		.094
Item 12	.935		.190		.089	.841		.290		.209
Item 13	.901			.365	.056	.773			.533	.118
Item 14	.877			.420	.053	.732			.597	.108
Item 15	.896			.397	.040	.764			.576	.085
Item 16	.887			.362	.081	.749			.521	.167
Item 17	.911			.365	.037	.793			.540	.080
Item 18	.891			.371	.069	.755			.535	.144
ω	.995	.924	.864	.939		.985	.924	.863	.940	

Note. λ: Factor loading; δ: Item uniqueness; ω: Omega coefficient of composite reliability; although these parameter estimates come from an invariant measurement model, invariance constraints are imposed on the unstandardized parameters estimates, so that standardized parameter estimates can still differ slightly across samples due to the partial invariance of the latent variance-covariance matrix; all coefficients are statistically significant ($p \leq .01$).

Table S3

Standardized Parameter Estimates for the Predictors and Outcomes' Measurement Model (Military Sample Only)

	Global Empowerment (λ)	Impact (λ)	Competence (λ)	Meaning (λ)	Autonomy (λ)	Turnover Intentions (λ)	Career Satisfaction (λ)	δ
Item 1	.605	.566						.383
Item 2	.544	.748						.075
Item 3	.598	.758						.068
Item 4	.330		.812					.231
Item 5	.348		.880					.105
Item 6	.355		.868					.121
Item 7	.369		.776					.263
Item 8	.750			.627				.044
Item 9	.730			.567				.146
Item 10	.782			.533				.104
Item 11	.607				.427			.449
Item 12	.232				.450			.741
Item 13	.322				.676			.422
Item 14	.813				.158			.314
Item 15	.572				.380			.529
Item 16	.387				.647			.368
Item 17						.749		.439
Item 18						.911		.170
Item 19						.809		.345
Item 20							.773	.400
Item 21							.769	.410
Item 22							.882	.223
ω	.941	.891	.939	.910	.726	.865	.850	

Note. λ: Factor loading; δ: Item uniqueness; ω: Omega coefficient of composite reliability; all coefficients are statistically significant ($p \leq .01$).

Table S4

Reliability and Correlations for the Variables used in this Study

	ω	α	1	2	3	4	5	6	7	8	9	10	11
Civilian Sample													
1. Global Fatigue (G)	.985	.960											
2. Physical Fatigue (S)	.924	.937	.152**										
3. Mental Fatigue (S)	.863	.949	.342**	-.205**									
4. Emotional Fatigue (S)	.940	.954	.139**	-.184***	-.254**								
Military Sample													
1. Global Fatigue (G)	.995	.981											
2. Physical Fatigue (S)	.924	.968	.178**										
3. Mental Fatigue (S)	.864	.977	.130**	-.176**									
4. Emotional Fatigue (S)	.939	.985	.097**	-.214**	-.504**								
5. PE Autonomy (S)	.726	.805	-.431**	-.062*	-.046	-.134**							
6. PE Meaning (S)	.910	.955	.109**	.082**	.043	.022	-.267**						
7. PE Impact (S)	.891	.917	-.029	-.056*	.094**	-.043	.051	-.294**					
8. PE Competence (S)	.939	.895	-.011	.005	-.013	.013	-.162**	-.04	-.047				
9. Global PE (G)	.941	.876	-.370**	-.027	.021	-.124**	.221**	.225**	.126**	.101**			
10. Career Satisfaction	.850	.790	-.498**	-.084**	.002	-.142**	.571**	-.017	.157**	-.075**	.659**		
11. Turnover Intentions	.865	.800	.495**	.053*	.034	.139**	-.481**	-.079**	.057*	.081**	-.613**	-.753**	
12. Job Satisfaction (SI)	n/a	n/a	-.426**	-.041	-.039	-.125**	.327**	.169**	.018	-.021	.692**	.624**	-.648**

Note. **p* < .05; ***p* < .01; ω: Omega coefficient of composite reliability; α: Alpha coefficient of scale score reliability; PE: Psychological empowerment; G: Global factor score from a bifactor measurement model S: Specific factor score for a bifactor measurement model; SI: Single indicator observed variable.

Table S5

Detailed Results from the Final Latent Profile Analytic Solution (Distributional Similarity)

	Profile 1		Profile 2		Profile 3		Profile 4		Profile 5		Profile 6 Military		Profile 6 Civilian	
	Mean	CI	Mean	CI	Mean	CI	Mean	CI	Mean	CI	Mean	CI	Mean	CI
Global	-1.016	[-1.022; -1.009]	-.253	[-.396; -.110]	-.200	[-.217; -.184]	.280	[.224;.336]	.702	[.694; .710]	.134	[.038; .230]	-.127	[-.200; -.054]
Physical	-.405	[-.567; -.243]	.788	[.533; 1.042]	-.545	[-.676; -.415]	-.054	[-.145; .037]	.01	[-.006; .026]	-.027	[-.096; .041]	-.145	[-.259; -.030]
Mental	-.482	[-.492; -.472]	-1.029	[-1.229; -.830]	-.631	[-.677; -.585]	.589	[.474; .703]	-.195	[-.217; -.173]	-.119	[-.162; -.075]	-.190	[-.309; -.071]
Emotional	-.067	[-.070; -.063]	.274	[-.010; .558]	.377	[.343; .411]	-.298	[-.412; -.184]	.633	[.613; .653]	.127	[.075; .179]	.156	[.089; .223]
	Profile 1		Profile 2		Profile 3		Profile 4		Profile 5		Profile 6 Military		Profile 6 Civilian	
	Variance	CI	Variance	CI	Variance	CI	Variance	CI	Variance	CI	Variance	CI	Varian ce	CI
Global	.000	[.000; .001]	.153	[.080; .226]	.000	[.000; .001]	.297	[.271; .323]	.001	[.001; .001]	.903	[.784; 1.022]	.172	[.144; .199]
Physical	.355	[.205; .506]	1.116	[.677; 1.555]	.015	[-.026; .056]	1.030	[.884; 1.176]	.005	[.003; .007]	.328	[.278; .379]	.471	[.342; .599]
Mental	.001	[.001; .001]	.313	[.120; .507]	.002	[-.001; .004]	.357	[.281; .433]	.007	[.004; .009]	.099	[.071; .127]	.230	[.186; .275]
Emotional	.000	[.000; .000]	.934	[.451; 1.418]	.001	[.000; .002]	.861	[.754; .969]	.004	[.002; .007]	.155	[.111; .198]	.327	[.277; .378]

Note. CI = 95% confidence interval*;* the profile indicators are estimated from factor scores with a mean of 0 and a standard deviation of 1, with the exception of the global fatigue factor for the civilian sample. Profile 1: Low Fatigue; Profile 2: Physically and Emotionally Depleted; Profile 3: Emotionally Depleted; Profile 4: Globally and Mentally Depleted; Profile 5: Globally and Emotionally Depleted; Profile 6: Balanced (Military and Civilians).

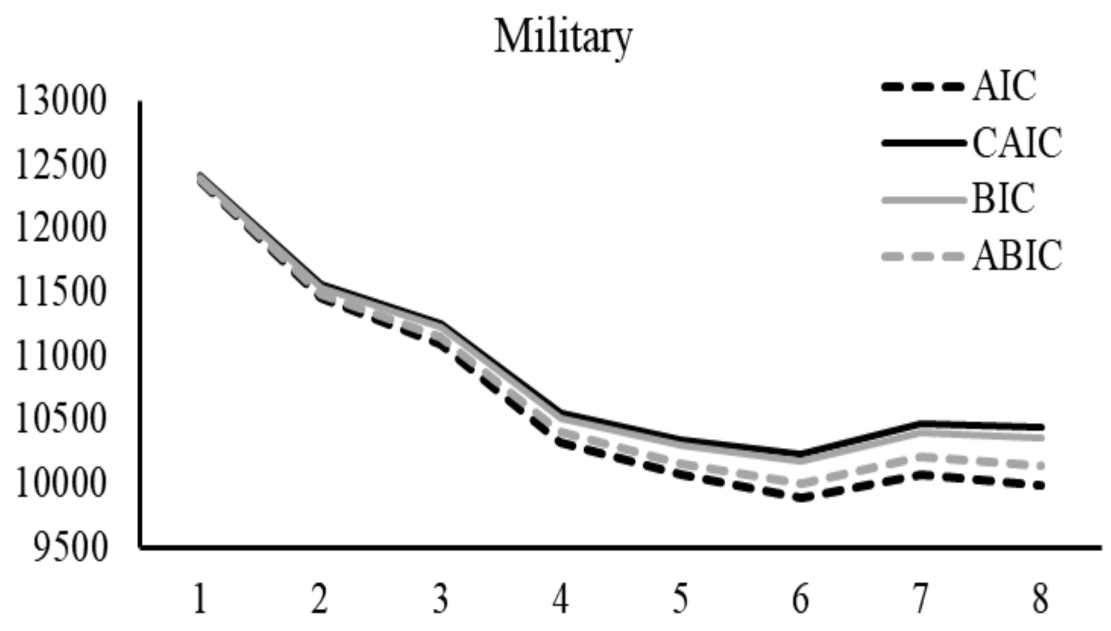

Figure S1. Elbow plot of the information criteria for the military sample latent profile analyses.

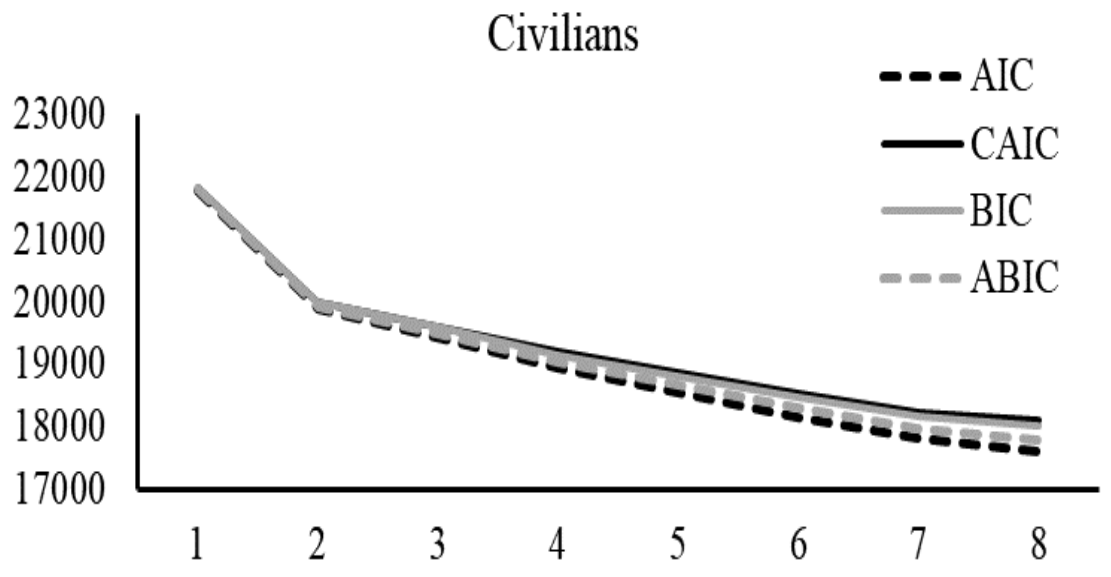

Figure S2. Elbow plot of the information criteria for the civilian sample latent profile analyses.
